# Effects of flow rate on the migration of different plasticizers from PVC infusion medical devices

**DOI:** 10.1371/journal.pone.0192369

**Published:** 2018-02-23

**Authors:** Lise Bernard, Teuta Eljezi, Hélène Clauson, Céline Lambert, Yassine Bouattour, Philip Chennell, Bruno Pereira, Valérie Sautou, on behalf of the ARMED Study Group

**Affiliations:** 1 Université Clermont Auvergne, CHU Clermont-Ferrand, CNRS, SIGMA Clermont, ICCF, Clermont–Ferrand, France; 2 CHU Clermont-Ferrand, Service Pharmacie, Clermont-Ferrand, France; 3 CHU Clermont-Ferrand, Direction de la Recherche Clinique et Innovation, Clermont-Ferrand, France; Bioinformatics Institute, SINGAPORE

## Abstract

Infusion medical devices (MDs) used in hospitals are often made of plasticized polyvinylchloride (PVC). These plasticizers may leach out into infused solutions during clinical practice, especially during risk-situations, e.g multiple infusions in Intensive Care Units and thus may enter into contact with the patients. The migrability of the plasticizers is dependent of several clinical parameters such as temperature, contact time, nature of the simulant, etc… However, no data is available about the influence of the flow rate at which drug solutions are administrated. In this study, we evaluated the impact of different flow rates on the release of the different plasticizers during an infusion procedure in order to assess if they could expose the patients to more toxic amounts of plasticizers. Migration assays with different PVC infusion sets and extension lines were performed with different flow rates that are used in clinical practice during 1h, 2h, 4h, 8h and 24h, using a lipophilic drug simulant. From a clinical point of view, the results showed that, regardless of the plasticizer, the faster the flow rate, the higher the infused volume and the higher the quantities of plasticizers released, both from infusion sets and extension lines, leading to higher patient exposure. However, physically, there was no significant difference of the migration kinetics linked to the flow rate for a same medical device, reflecting complex interactions between the PVC matrix and the simulant. The migration was especially dependent on the nature and the composition of the medical device.

## Introduction

In the field of infusion, numerous medical devices, such as infusion sets and extension lines are used in various simple or complex assemblies. Most of them are manufactured in PVC plasticized with alternatives to di-(ethylhexyl)-phthalate (DEHP) plasticizers, e.g. di-(ethylhexyl)-terephthalate (DEHT), di-isononyl-1,2-cyclohexane-dicarboxylate (DINCH) or trioctyl trimellitate (TOTM) and diisononyl phthalate (DINP). They have greatly replaced DEHP since it is likely to present a danger to the patient, and has now been classed as a CMR1b risk substance due to its effects on reproduction and fertility [1]. Thus, it now must not exceed 0.1% by mass of the plasticized material, as defined by the European regulation concerning the Registration, Evaluation, Authorization and Restriction of Chemical substances (REACH). Furthermore, according to decree of 13^th^ April 2017, its use in tubings used in pediatric, neonatal, and maternity units has been restricted in France above a threshold level of 0.1% [2]

However, only very limited data is available regarding the risk associated to the migration of the alternative plasticizers from the medical devices, especially in at risk-situations like multiple infusions in Intensive Care Units (ICU). To assess the exposure risk of inpatients, the evaluation of their migration in such conditions is required. An infusion model was developed to estimate if a medical device could be considered safe for infusion use according to the leaching of plasticizers [3]. Nevertheless, this model doesn’t take into account of the flow rate at which drug solutions are administrated to the patients, thus considering the exposure risk to be the same whatever the flow rate of the infusion. Bagel et al demonstrated that the extraction of DEHP was encouraged in static conditions [4] but no study has evaluated the influence of this parameter with alternative plasticizers.

The aim of this work was to evaluate the impact of different flow rates on the release of four plasticizers (TOTM, DINP, DEHT and DINP) during an infusion and to assess different infusion rates that could expose the patient to more toxic amounts of plasticizers. From this basis, it could be discussed whether the infusion model should be adapted and eventually if reduction factors linked to the flow rate should be applied, correcting the specific migration with low or high flow rates (as it has been done in the regulation 10/2011 [5] with food containing more than 20% fat).

To this end, we tested different flow rates usually applied in clinical practice through infusion sets and extension lines. This study should finally help us to understand which mechanisms govern migration phenomena of alternative plasticizers.

## Materials and methods

### Samples

MDs used for the migration assays were extension lines and infusion sets, which are commonly used in the field of infusion. They were selected specifically because they contain one specific different plasticizer each (TOTM, DEHT, DINCH or DINP) without any trace of carcinogenic, mutagenic, reprotoxic 1b (CMR1b) phthalates, as announced by their manufacturer. To allow correct comparability, each device was also chosen so as to have the same technical features in terms of the tube thickness (which is approximately 0.75mm for the extension lines and 0.6mm for the infusion sets).

The characteristics of the chosen medical devices are presented in Tables 1 and 2.

**Table 1:** characteristics of PVC tubings from extension lines used in the migration study.

Supplier	Cair LGL	Codan	B Braun	Sendal	Cair LGL	Cair LGL
**Reference**	PES 3301 M	E-87 P	0086670 D	Prolonsend	PN 3301 M	PN 3101 M
**Batch**	15D13T	H71654-1	14N02F8SPA	03446	13E21-TN	12H07-TN
**Designation**	EL 1	EL 2	EL 3	EL 4	EL 5	EL 6
**Length (cm)**	13.9	96.5	11.2	11.0	10.2	10.5
**Weight of PVC tubing (mg)**	1286.8	2837.8	1050.4	893.1	992.0	506.8
**Inner diameter (cm)**	0.25	0.10	0.25	0.30	0.25	0.10
**Internal surface (cm^2^)**	10.91	30.30	8.79	10.36	8.01	3.30
**Volume (mL)**	0.682	0.757	0.550	0.777	0.500	0.082
**Co-extruded (PVC/PE)**	Yes	No	No	No	No	No
**Announced plasticizer**	**TOTM**	**TOTM**	**DEHT**	**DINP**	**DINCH**	**DINCH**

**Table 2:** characteristics of PVC tubings from infusion sets used in the migration study.

Supplier	B Braun	CareFusion	Doran International	Codan
**Reference**	4063007	A64	INFU-R3	43.4535
**Batch**	039615B13A8421	0396	1411247	L85603-1
**Designation**	IS 1	IS 2	IS 3	IS 4
**Length (cm)**	179.0	145.7	181.7	173.8
**Weight of PVC tubing (mg)**	12975.5	11470.7	15052.2	12258.5
**Inner diameter (cm)**	0.27	0.27	0.25	0.30
**Internal surface (cm^2^)**	154.6	124.4	141.5	170.3
**Volume (mL)**	10.4	8.5	8.8	13.3
**Co-extruded (PVC/PE)**	No	No	No	No
**Announced plasticizer**	**DEHT**	**DINP**	**DINCH**	**TOTM**

### Migration assays

#### Analysis of plasticizers in PVC tubings

Before performing migration tests, the exact composition of plasticizers present in each PVC tubing was determined by GC-MS after a solvent extraction using the published method described by Bourdeaux et al [6].

This analysis allowed us to identify and quantify the main plasticizer present in the PVC matrix as well as the minority ones, which could be found as impurities and could also be released from the devices.

#### Conditions of the migration assays

Migration assays were performed in the same conditions described by Bernard et al as regard to the choice of the simulant, and of the contact temperature and time [3]. Only the flow rate was modified.

The used parameters were as follows:

- simulant: we chose a 50/50 ethanol/water (v/v) solution which reflects lipid emulsions able to extract plasticizers from PVC medical devices. 3% acetic acid (also proposed in the infusion model) was not selected because the migration appears to be insignificant in it [3].
- contact temperature: 25°C was chosen to perform the tests as it corresponds to the ambient temperature most commonly found in adult health care units.
- contact time: the infusion tests were performed during 24h in order to mimic the maximal time period of an injectable drug infusion containing lipids.

#### Administration methodology

Based on clinical practices, assays were performed under different flow rates that are usually applied from a syringe pump for the administration of drugs and on infusion sets for gravity administrations. The conditions were the following:

- for the tests with extension lines, the syringes were filled with the ethanolic simulant and the extension line was set on the syringe. Then one of the following flow rates was applied during 24 hours: 1mL/h, 5mL/h and 10mL/h
- for the tests with infusion sets, non-PVC bags were filled with the ethanolic solution and set on the infusion set. Then one of the following flow rates was applied during 24 hours: 8mL/h, 20mL/h, 50mL/h and 100mL/h

The 8mL/h flow rate was specifically chosen since it is in accordance with the clinical ratio volume/surface of 2L/13dm^2^ recommended in the infusion model of Bernard et al. (all the infusion sets tested in this work have similar inner surface in contact with simulant). The other flow rates correspond to those usually set up in clinical practice: the lower ones with extension lines allow the continuous administration of narrow therapeutic range drugs (e.g amines, anaesthetics, insulin, etc…) whereas the higher ones are used for injectable chemotherapy or antibiotherapy drugs.

#### Kinetic study

In order to study the migration mechanisms, we performed a kinetic study by analysing the cumulated amount of plasticizers released into the simulant at different contact times: 1h, 2h, 4h, 8h and 24h. For each contact time, assays were performed in triplicate.

#### Analysis of plasticizer into the simulant

The amount of plasticizers released into the simulant was assessed by gas chromatography coupled with mass spectrometry (GC-MS) [6] after extraction from the simulant. To perform this extraction, 600 μl of the ethanolic solution was taken and added to 600 μl of a 2 μg/mL of (benzylbutylphtalate) BBP solution in chloroform. After homogenization (with vortex 20 Hz, 30 seconds), the samples were centrifuged (3500 rpm, 5min). Finally, the chloroform phase (below the aqueous phase) was taken for GC-MS analysis.

As plasticizers are widely present in the environment, to prevent the risk of contamination, the used glass flasks were washed 3 times with chloroform before performing the assays; hemolysis tubes and GC-MS vials were single use.

### Expression of the results

- The initial amount of each plasticizer in the tubing samples was expressed in mass percent (%) (mean ± standard deviation)
- Two different approaches were undertaken to verify the impact of the flow rate on the release of the plasticizers:
  - a “clinical approach”which gives the amounts of plasticizers able to migrated to patients. The amounts of each plasticizer released into the simulant were expressed in two manners:
    - the mass (μg) of the plasticizer released into the simulant
    - this amount was then compared with the initial weight of the PVC sample (mg). It is expressed in mg of migrated plasticizer per 100g of PVC
  -a “physicochemical approach” which provides the results of release by standardizing the features of the MD and the volume infused. The migration kinetic was expressed as follow:
    A=q÷(s×v)
    A = migration kinetic (μg/dm^2^/mL)
    q = quantity of plasticizer released into the simulant (μg) during the migration assay
    s = area of MD in contact with the simulant (dm^2^) during the migration assay
    v = infused volume during the migration assay (mL)

### Statistical analysis

All statistical analyses were performed using Stata statistical software (version 13, StataCorp, College Station, US). The tests were two-sided, with a type I error set at α = 0.05. Continuous parameters were expressed as mean ± standard-deviation according to statistical distribution (assumption of normality studied using Shapiro-Wilk’s test). To study longitudinal evolution, correlated repeated data was analyzed using linear mixed models: (1) quantity of plasticizers released during migration assays from the extension lines and from the infusion tests and (2) kinetic of migration of plasticizers. This approach seems more relevant rather than usual statistical tests because assumption concerning independence of data is not met. The (fixed) effects group, time-point evaluation and their interactions time/flow rate were studied. The normality of residuals obtained from these models was analyzed as described previously. Bayesian Information Criterion (BIC) was estimated to determine the most appropriate model, notably concerning the covariance structure for the random-effects due to repeated measures across the time and consequently to the autocorrelation.

This approach seems more relevant rather than usual statistical tests because assumption concerning independence of data has not been reach met

## Results

All raw data are given in S1 File.

### Plasticizers in PVC tubings

Table 3 shows the nature and the amount of plasticizers in each PVC medical device as analyzed by GC-MS.

**Table 3:** Qualitative and quantitative composition in plasticizers of the studied medical devices.

Type of PVC medical device	Medical device (MD)	Nature of plasticizer in MD	Quantity of plasticizer in MD (expressed in mass percent)
Extension line	EL n°1 (Cair)	TOTM	31.81
		DEHT	0.06
		DEHP	<LOQ*
	EL n°2 (Codan)	TOTM	30.29
		DEHT	0.05
		DEHP	<LOQ*
	EL n°3 (BBraun)	DEHT	26.74
	EL n°4 (Sendal)	DINP	48.72
		DEHP	0.25
	EL n°5 (Cair GM)	DINCH	30.16
	EL n°6 (Cair PM)	DINCH	35.73
Infusion set	IS n°1	DEHT	37.50
	IS n°2	DINP	34.89
	IS n°3	DINCH	44.27
		DEHP	0.05
	IS n°4	TOTM	40.97
		DEHT	0.15
		DEHP	0.002

* LOQ = limit of quantification;

EL = extension line; IS = infusion set.

### Plasticizers migration during infusion: The clinical approach

**Fig 1:**
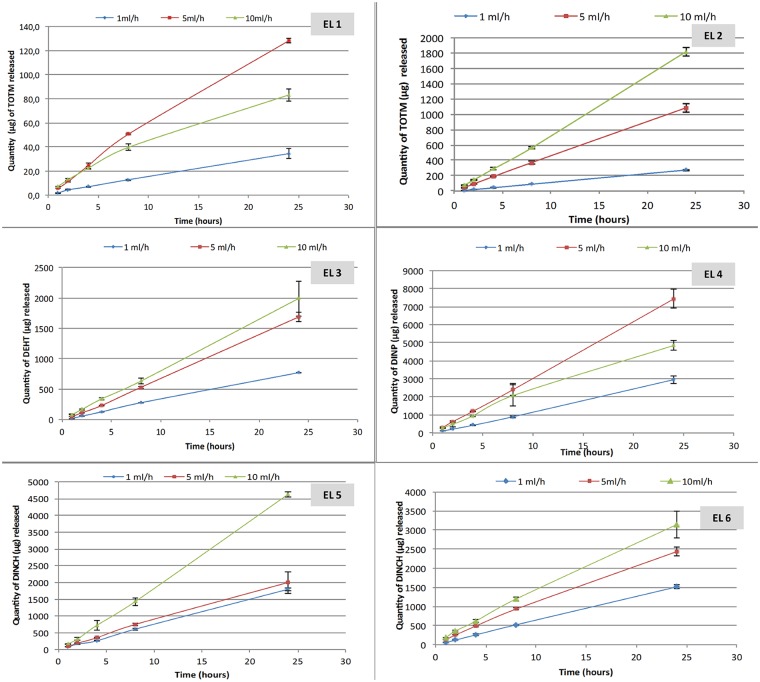
Quantity of plasticizers released during migration assays from the 6 extension lines (EL) tested (n = 3; mean +/- standard deviation).

**Fig 2:**
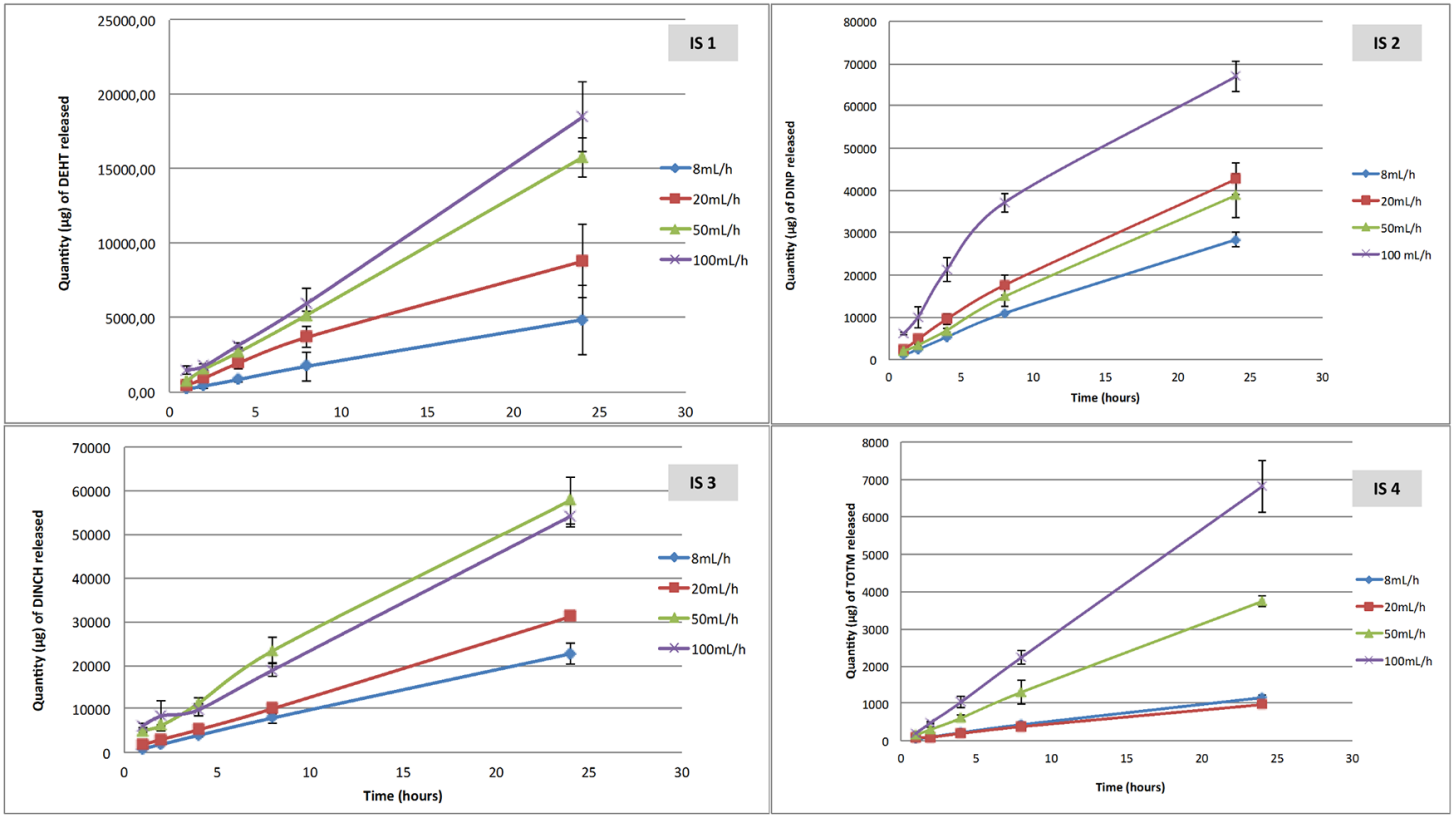
Quantity of plasticizers released during migration assays from the 4 infusion sets tested (n = 3, mean +/- standard deviation).

Figs 1 and 2 show the results of the migration assays of the main plasticizers from the 6 tested extension lines and the 4 infusion sets.

The quantity of plasticizers released into the simulant raises gradually during the infusion period, regardless the type of device and the plasticizer integrated into the PVC.

The amounts of plasticizers released at the end of the procedure (24h) are different according the flow rate: the higher the flow rate, the higher the infused volume and the higher the quantities of plasticizers released, both from infusion sets and extension lines.

For examples, by comparing the highest and the lowest flow rates in both migration studies (100mL/h vs 8mL/h with infusion sets and 10mL/h vs 1mL/h with extension lines), the ratios of the amounts of plasticizers released at each flow rate are 6.9 and 8; 3.6 and 2.7; 3.35 and 2.6 and 2.3 and 1.7 for TOTM, DEHT, DINCH and DINP respectively.

Regarding both extension lines of PVC/TOTM, TOTM migration is more important when the device doesn’t possess an internal layer of polyethylene. The quantities of TOTM released are about ten times higher than those released from the coextruded extension line, regardless of the flow rate.

The inner diameter of the tubing does not have any impact on the migration of DINCH. Indeed, the total amounts of DINCH released from the EL5 and the EL6 are quite similar (for an inner diameter of 0.25 and 0.1 cm, respectively) whatever the kinetic time.

**Fig 3:**
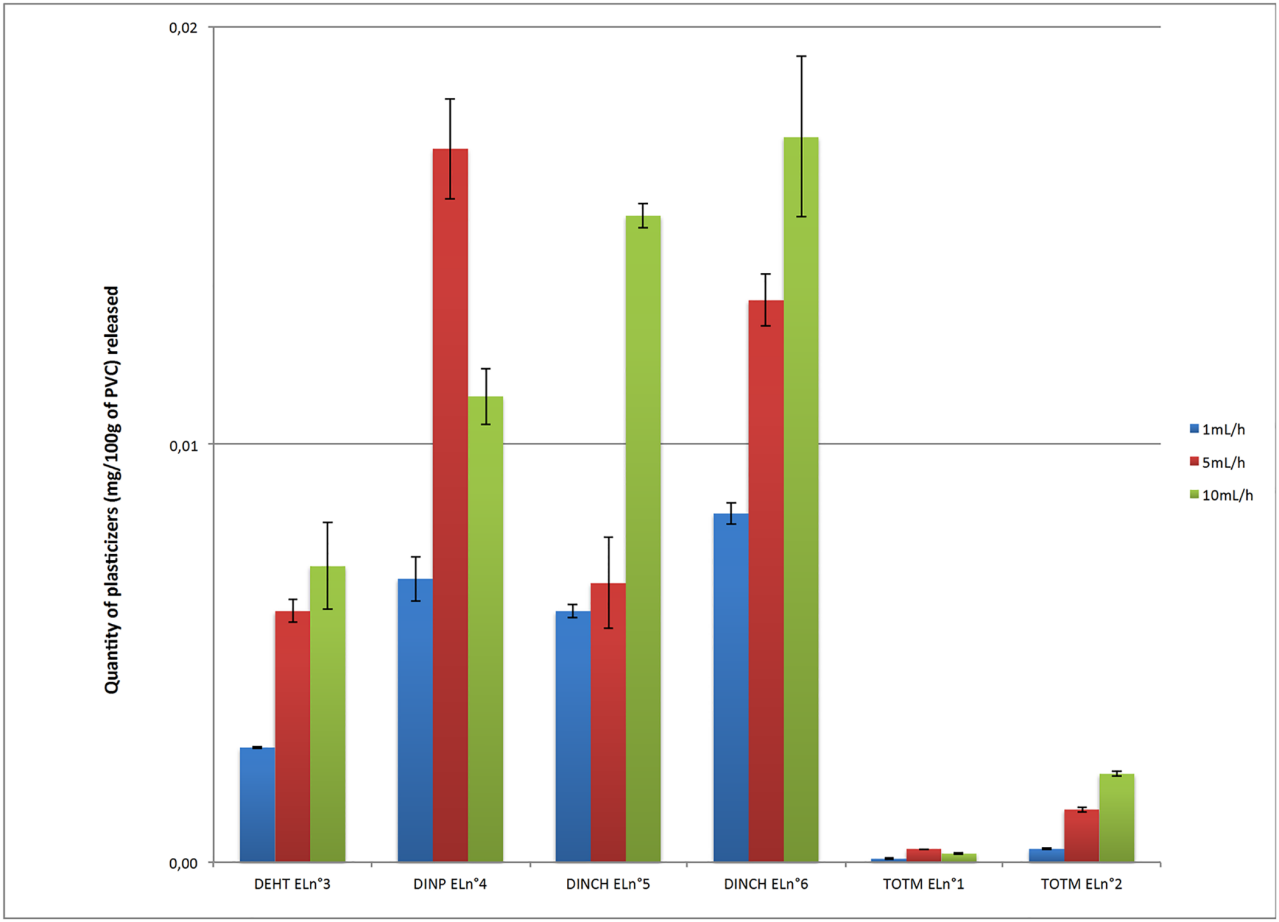
Comparison of the migration of plasticizers from the extension lines at 24h according the flow rate (n = 3, mean +/- standard deviation).

**Fig 4:**
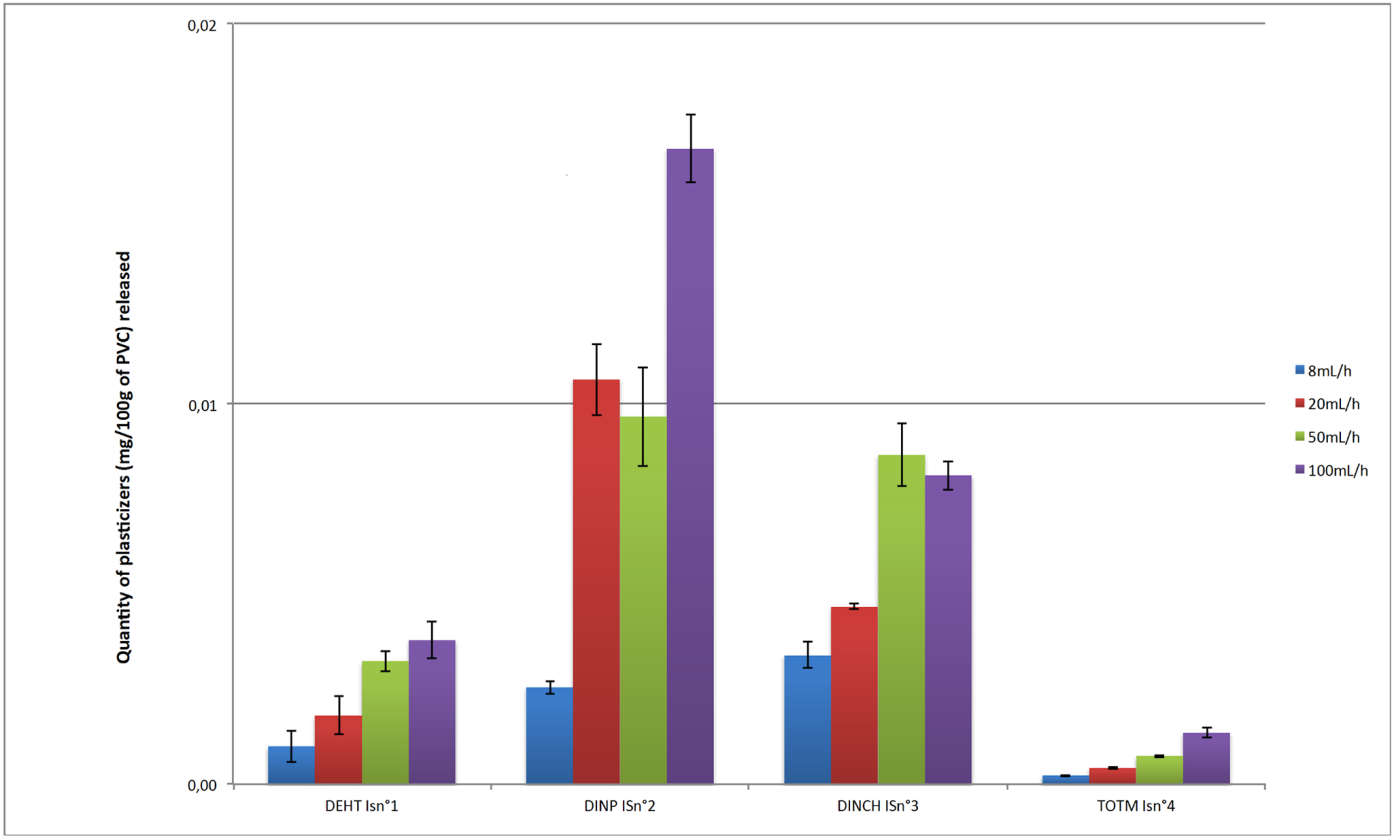
Comparison of the migration of plasticizers from the infusion sets at 24h according the flow rate (n = 3, mean +/- standard deviation).

Figs 3 and 4 show the percentages of the initial amounts of DEHT, TOTM, DINCH and DINP having migrated from the extension lines and the infusion sets at 24h as a function of the flow rate set.

Under identical dynamic experimental conditions, the plasticizers demonstrated different migration abilities within the first 24 h of contact. Regardless the flow rate, DINP and DINCH had the highest degrees of migration in dynamic conditions compared to TOTM and DEHT.

### Plasticizers migration during infusion: The physicochemical approach

**Fig 5:**
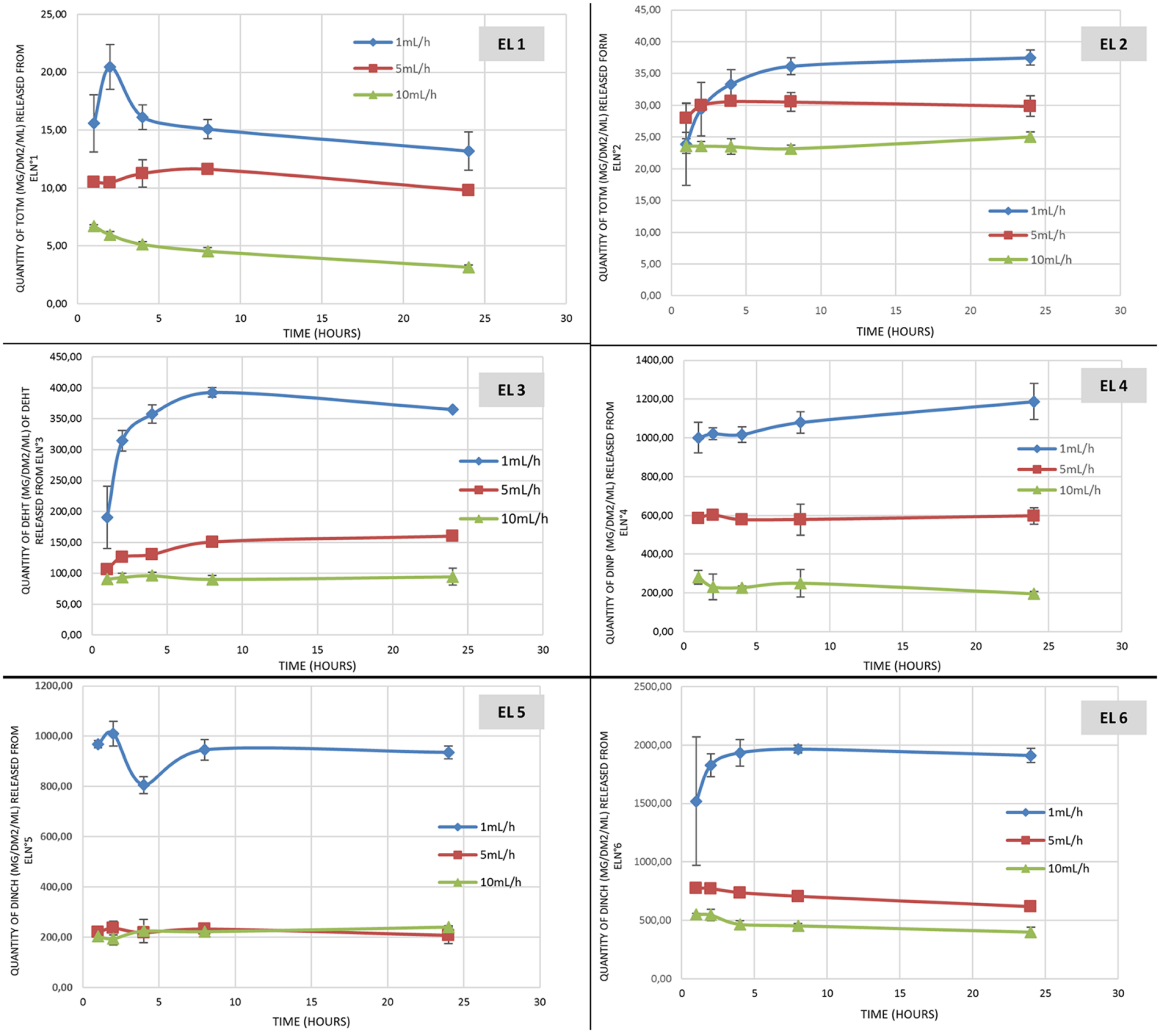
Kinetics of the plasticizer’s migration from the 4 extension lines (n = 3, mean +/- standard deviation).

**Fig 6:**
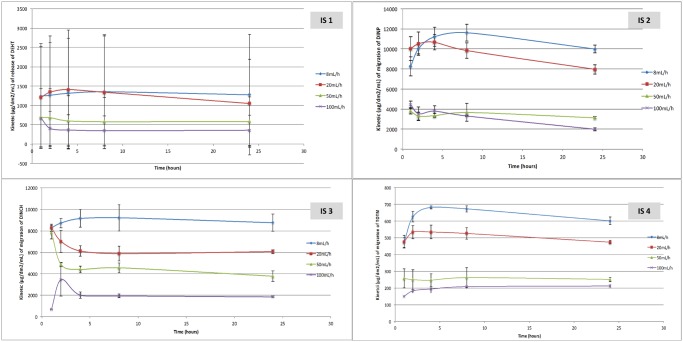
Kinetics of the plasticizer’s migration from the 4 infusion sets (n = 3, mean +/- standard deviation).

Figs 5 and 6 show the quantities of the different plasticizers released from the MD expressed by unit area of MD in contact with the simulant (dm^2^), by unit volume infused (mL) during the infusion procedure. The application of a lower flow rate may extract more plasticizer at each time of the kinetic, regardless of the plasticizer and the type of device. The findings of statistical analyses reported in Tables 4 and 5 are thus consistent except for DEHT from the infusion sets for which results show an important variability.

**Table 4:** Statistical analysis of the impact of the flow rate on the migration profile for each plasticizer: (part A) study of the interaction time/flow rates and (part B) comparisons between flow-rates at any time from the extension lines. Results were expressed as p-values.

	DEHT	TOTM(EL 1)	TOTM (EL 2)	DINCH (EL 5)	DINCH (EL 6)	DINP
**A**						
**Time/flow rate at time 2h**	0.17	0.001	0.04	0.89	0.54	0.49
**Time/flow rate at time 4h**	0.07	0.20	<0.001	0.63	0.33	0.50
**Time/flow rate at time 8h**	0.02	0.28	<0.001	0.91	0.29	0.31
**Time/flow rate at time 24h**	0.05	0.44	<0.001	0.85	0.29	0.01
**B**						
5mL/h vs 1mL/h	<0.001	0.125	0.008	<0.001	<0.001	0.006
10mL/h vs 1mL/h	<0.001	0.015	0.001	<0.001	<0.001	0.005
10mL/h vs 5mL/h	0.010	<0.001	0.040	0.392	0.007	0.003

**Table 5:** Statistical analysis of the impact of the flow rate on the migration profile for each plasticizer: (part A) study of the interaction time/flow rates and (part B) comparisons between flow-rates at any time from the infusion sets. Results were expressed as p-values.

	DEHT	TOTM	DINCH	DINP
**A**				
**Time/flow rate at time 2h**	0.78	0.47	0.11	0.45
**Time/flow rate at time 4h**	0.71	0.35	0.43	0.35
**Time/flow rate at time 8h**	0.70	0.47	0.42	0.30
**Time/flow rate at time 24h**	0.82	0.87	0.41	0.38
**B**				
20mL/h vs 8mL/h	0.96	<0.001	<0.001	0.44
50mL/h vs 8mL/h	0.13	<0.001	<0.001	<0.001
100mL/h vs 8mL/h	0.05	<0.001	<0.001	<0.001
50mL/h vs 20mL/h	0.27	<0.001	0.02	<0.001
100mL/h vs 20mL/h	0.11	<0.001	<0.001	<0.001
100mL/h vs 50mL/h	0.66	0.02	<0.001	0.95

However, except for the non-coextruded extension line (EL2), Tables 4 and 5 show that the migration kinetic profiles of each plasticizer are similar at any time, without any significant difference in the interactions between time and flow rate; in other words the variation over time was not significantly different between pla**s**ticizers.

As with the « clinical approach », TOTM migration, expressed in mg/dm^2^/mL, is different between PVC tubings and PVC/PE tubings. TOTM release is 5 times, 3 times and 2.5 times lower from the coextruded extension line (EL 1) than that from the non-coextruded one (EL 2) at respectively 10mL/h, 5mL/h and 1mL/h.

## Discussion

This study has assessed, for the first time, the impact of the flow rate on the migration of DEHP alternative plasticizers from medical devices, using two different approaches that are complementary to characterize this influence.

With the clinical approach, the flow rate does influence the migration of all tested plasticizers, by increasing the released plasticizer levels in concordance with an increased infusion rate, leading to higher amounts of plasticizers delivered to the patient after a 24h-period infusion procedure. Each plasticizer has a specific migrability, as demonstrated in our previous work [7]: in the same conditions, TOTM was found to have the lowest migration rate, followed by DEHT and then DINCH. We also studied DINP’s migration in this study, which is close to that of DINCH: at 24h, respectively 1.10% and 1.67% of initial amounts of DINP had been extracted from the extension line and the infusion set at the highest flow rates of 10mL/h and 100mL/h compared to the 1.50% (bigger diameter) and 0.82% of DINCH found in the simulant. For TOTM and DEHT, the extracted levels were much lower: 0.02% (coextruded line) and 0.14% for TOTM and 0.70% and 0.40% for DEHT at 24h.

Comparisons with other studies assessing the migration of plasticizers under dynamic conditions could be performed. Most of them have been carried out with DEHP. In the work of Bagel et al, 3 different flow rates were investigated [4]: 30, 60 and 90 mL/h. For a same infused volume by extension lines, the authors showed that about 1000 μg of DEHP is released in 8.33h at 30 mL/h, 600 μg in 4.17h at 60 mL/h and 500 μg in 2.8h at 90 mL/h. In our study, higher levels of DINP (respectively 17610 μg, 6872 μg and 9961 μg), DINCH (respectively 10060.9 μg, 11285.9 μg and 8393.3 μg), and DEHT (respectively 3687.9 μg, 2672.4 μg and 1745.3 μg) were found in the simulant after 8h at 20 mL/h, 4h at 50 mL/h and 2h at 100 mL/h. The quantities of TOTM released were much lower at the same times and speeds: 378.8 μg, 609.1 μg and 489.1 μg. The same comparisons could be performed with the work of Rose et al, showing a 500 μg release of DEHP in a propofol solution and 300 μg in an intralipid solution at 12 mL/h at 24°C during 6h [8]. Our results concerning plasticizer migration levels at 10 mL/h after 8h of infusion are the following: 2071.1 μg of DINP, 1425.8 μg and 11935 μg (large and small diameter) of DINCH, 632.5 μg of DEHT, 40 μg and 561.6 μg (coextruded and non-coextruded tubing) of TOTM. The amounts of TOTM are even higher after 4h of dialysis session at 500 mL/h (Kambia et al 2001). All this data is consistent with our study to show that the flow rate influences the leaching of all the plasticizers to various degrees depending of the nature of the plasticizer and the simulant. The highest quantities of additives released in the media are observed with highest volumes of simulant infused, whereas the migration is more important with lowest flow rates for a same infused volume. However, data comparison is difficult, because the experimental conditions are not standardized between the studies in terms of nature and volume of simulant, flow rates, contact time, temperature, etc… Moreover, in the field of infusion, two main types of medical devices are used: infusion sets and extension lines that could have a different influence on the release of plasticizers from the PVC matrix. Our work allows this comparison, by assessing in standardized conditions different types of infusion devices made of plasticized PVC, simulating the general clinical practice in adult and pediatric care units. The data collected demonstrates that the flow rate plays a role in the migration of plasticizers. The migration kinetic is higher when drugs are infused at lower flow rates but for a given time period, the greater the flow rate, the higher the level of plasticizer released in the simulant at 24h. This could be easily explained by the volume infused of the simulant. These differences may have important clinical implications: patients receiving such IV therapies may be exposed to variable doses of plasticizers that ideally should remain under the tolerable limit before toxicity, i.e the DNEL (Derived No Effect Level) which represents the human theoretical dose limit. Considering the largest amounts of plasticizers quantified in the simulant at 24h (i.e. at a flow rate of 100mL/h), an inpatient of 70 kg is thus susceptible to be exposed to 0.097mg/kg/d of TOTM, 0.264mg/kg/d of DEHT, 0.77mg/kg/d of DINCH and 0.96mg/kg/d of DINP. For DINP, the obtained value is above the TDI (Tolerable Daily Intake) of 0.15 mg/kg/d of the Danish EPA report [9] For DINCH, the value remains beneath the TDI of 0.15 mg/kg/d of the Danish EPA report or the latest NOAEL limit of 300mg/kg/d for an IV route administration of DINCH given by the work of David et al [10], considering a DNEL of 3mg/kg/d (security factor of 100). However, the exposure dose could be considered as toxic, when compared to the 0.4 mg/kg/d of the NICNAS report [11]. For TOTM, as expected, exposure doses are the lowest of all the plasticizers and are far below the TDI of 1.13mg/kg/d [9].

Nevertheless, to perform a risk assessment according to the flow rate, the entire infusion therapy should be considered, and not just one infusion procedure per patient. The physicochemical approach could provide information on the specific migration rates of each PVC medical device. The migration kinetic is higher when the solvents are infused at lower flow rates, with major differences between extension lines and infusion sets. The ratios of migration kinetics between the low and the high flow rate for extension lines were of 8 (non coextruded MD) and 2.3 (coextruded MD) for TOTM, 2.7 for DEHT, 2.1 and 2.6 for DINCH and 1.7 for DINP.

When using the physicochemical approach, the quantities of released plasticizers were compared by MD surface unit and infused volume in order to assess the real impact of the flow rates on the different types and models of our studied medical devices. We showed that there was no significant difference of the migration kinetic profiles depending on the flow rate for a same medical device. Notably, quantities of DEHT released were the same whatever the time of kinetic, which suggests an atypical behavior of the plasticizer.

These results highlight that the migration of plasticizers is very variable depending on the type of medical devices:

- type and use: the infusion sets are especially used for the administration of high volumes of drug solutions by gravity whereas the extension lines allow a more controlled and precise administration of only tens of milliliters of emergency drugs including some with a narrow therapeutic range. Moreover, the different administration positions (i.e vertical position for the infusion sets and horizontal for the extension sets) may influence the migration by influencing the surface interactions between the plasticizers and the simulant. The results highlight that the migration of plasticizers varies greatly with the type of medical device. Indeed, the quantities released at 24h for a flow rate of 10 mL/h with an extension line made of non-coextruded PVC/TOTM were of 25 μg/dm^2^/mL whereas they reached 600 μg/dm^2^/mL at 24h for a flow rate of 10mL/h with an infusion set made of non-coextruded PVC/TOTM, i.e a ratio of 24. For the other plasticizers, the same ratios are 18.8; 13.7 and 5 for DINCH, DEHT and DINP respectively.
  Moreover, a greater variability occurred during the migration assays with infusion sets versus extension lines. The main reason for this is that the flow rate was set up manually for the infusion sets unlike the automatic syringe pump administrations used for the tests using the extension lines.
- diameter: the migration kinetic of DINCH is almost 2 times lower when the inner diameter is 2.5 times higher (EL5 vs EL6), although a higher volume of simulant (with the larger diameter of the MD) does finally extract a higher quantity of plasticizer (see Result section Fig 5). This might be explained by different plasticizer/solvent interactions with the inner surface of the PVC and thus by the matrix penetration of the solvent. Indeed, it has been demonstrated that the storage conditions (i.e the simulant in contact with the PVC surface) could impact the characterization of the surface physico-chemistry, especially during a contact with ethanol. In the work of Salloum et al [12], the infusion MD surfaces are modified and even altered by the solvent during storage. The roughness of the surface, the size and the distribution of cracks on the surface are variable and depend on the type and composition of the MD and the duration of the contact. They showed that it leads to different leaching rates of the additives. In our study, the interactions between the ethanolic simulant and the PVC surface of the two extension lines containing DINCH as main plasticizer are different because of the specific changes on the surface occurring during the assays according the flow rate (different contact time) and the manufacturing process of the MDs.
- composition of the PVC matrix: we clearly demonstrated that the polyethylene layer has a protect effect on the migration of TOTM, lowering its migration rate by more than 2 times. As it has already been shown that coextruded extension lines avoid the drug sorption on PVC [4],[13], such devices should be used first in clinical practice. Moreover, all the medical devices tested in our work are composed of plasticized PVC with different initial quantities of plasticizers: extension lines contain 26.7% of DEHT, 31.8% (coextruded) and 30.3% (non coextruded) of TOTM, 30.6% (large diameter) and 35.7% (small diameter) of DINCH and 48.7% of DINP; whereas the infusion sets contain 37.5% of DEHT, 41% of TOTM, 44.3% of DINCH and 34.9% of DINP.

These differences should be linked to the higher variability observed with DEHT released from the infusion set. They probably also rely on the specific technicality of the gravity infusion set made of PVC/DEHT (provided by BBraun), although generally, more variability occurred during the migration assays with infusion sets versus extension lines. Moreover, the lower leaching aptitude into oily media of DEHT (i.e ethanolic simulant) compared to other studied plasticizers [14] could have influenced the interactions between DEHT and the simulant. To check this assumption regarding the infusion set supplied by BBraun (Intrafix Safeset), some additional migration assays were performed (see S2 File). The results confirm a higher variability rate due to the gravity technique (no variability appeared with the same tubing during an infusion with a syringe pump) and furthermore, provided particularly by this infusion set (less variability was observed with another infusion set).

Overall, these results suggest that the migration rate is less influenced by the flow rate than by the nature and the composition of the medical device, reflecting complex interactions between the PVC matrix and the simulant, combined with the volume of the infused drug to patient. This may explain a different diffusion ratio for each plasticizer inside the PVC matrix, as it has been demonstrated by Al Salloum et al, by using a coupling Raman confocal microscopy to UV spectroscopy technique [15]. We thus recommend that the flow rate be taken into account in the risk assessment of the plasticizers’ migration if the tested MD are likely to be used in conditions significantly different in terms of flow rate (which may lead to significant differences in the technical characteristics of tested MD).

## Conclusion

This study provides information about the real influence of the flow rate on the migration of plasticizers from PVC medical devices used in infusion conditions. The two approaches developed in our study complementarily characterize this influence. From a clinical view, higher speeds led to higher amounts of plasticizers released to inpatients for a fixed contact time of 24h. On the other side, from a physicochemical view, there was no significant difference of the migration kinetic in relation to the flow rate at each contact point of a same medical device. An increased consideration to the specific features of a medical device should be given, in order to assess patients’ exposure risk to alternative plasticizers. The different PVC tubing tested in this study are given as examples and reflect that the risk evaluation should be completed, including all the specific environment parameters. More information could be obtained by profile analyses of the PVC matrix in order to understand specific migration mechanisms according to the composition of the PVC matrix, which could help to develop solutions to prevent these surface interactions.

## Supporting information

S1 File Raw data of migration assays. (XLS)

S2 File Additional migration assays. (DOCX)
