# Supplementary material for: Effects of flow rate on the migration of different plasticizers from PVC infusion medical devices
Source: PLoS One. 2018 Feb 23;13(2):e0192369. doi: 10.1371/journal.pone.0192369 (PMC5825016; doi:10.1371/journal.pone.0192369)
Supplement: S2 File — (DOCX) [file pone.0192369.s002.docx]

ADDITIONAL MIGRATION ASSAYS

**METHODOLOGY**

**Samples**

MDs used for the additional assays were 2 of the infusion sets tested in the main study:

- Intrafix Safeset (supplied by BBraun): made of PVC/DEHT

- A64 (supplied by Carefusion): made of PVC/DINP

The references of both MD are:

| **Type** | **Reference** | **Batch number** | **extension life** | **Supplier** |
| --- | --- | --- | --- | --- |
| Infusion set A64 | A64 | 5513 | 30/09/21 | Carefusion |
| Infusion set Intrafix Safeset | 4063007 | 17L04A8421 | 31/10/19 | Bbraun |

**Conditions of the migration assays**

Migration assays were performed in the same conditions described in the main study as regard to the simulant (50/50 ethanol/water (v/v) solution), and the contact temperature (25°C)

The flow rate was set up at 20 mL/h: it was chosen among the flow rates used in the main study for the assays with infusion sets and because it also can be set up on a syringe pump.

The contact time was of 4 hours in order to have enough time to make regular kinetic sampling

**Administration methodology**

- gravity administration: non-PVC bags (the same bags that were used in the main study) were filled with the simulant. The 20mL/h flow rate was applied during 4 hours, and verified (by drop counting) regularly (at each sampling time)

- syringe pump administration: only the Intrafix Safeset was tested by this procedure, in order to compare the DEHT migration between both administration techniques. The tubing of the infusion set was cut and set on the syringe which was pre-filled with the simulant

**Kinetic study**

We performed a kinetic study by analysing the cumulated amount of plasticizers released into the simulant at different contact times: 30 min, 1h, 2 h, and 4 h. For each contact time, assays were performed in triplicate.

**Analyses**

- Comparison of the migration of DEHT by 2 infusion administration techniques (gravity and with a syringe pump):

The amount of plasticizers released into the simulant was assessed by the same analytical method used in the main study: gas chromatography coupled with mass spectrometry (GC-MS) after extraction from the simulant. The results obtained in μg/mL were then expressed in mg/dm^2^

- Comparison of the flow rates during gravity infusion with 2 different infusion sets provided by 2 different companies (Intrafix Safeset provided by BBraun and A64 provided by Carefusion): the mass of the simulant collected at each sampling time was measured (with a precision balance) and the mass gain vs T0 was calculated and expressed in %

**RESULTS**

**Syringe pump administration (Infusion set Intrafix Safeset, BBraun)**

|  |  | mean | standard deviation | variation coefficient |
| --- | --- | --- | --- | --- |
| DEHT concentration (mg/dm^2^) | T30min | 0.269 | 0.029 | 10.67% |
|  | T60min | 0.547 | 0.031 | 5.71% |
|  | T120min | 1.157 | 0.109 | 9.42% |
|  | T240min | 2.381 | 0.137 | 5.75% |
| Mass of simulant collected (g) | T30min | 9.170 | 0.549 | 5.99% |
|  | T60min | 17.237 | 0.526 | 3.05% |
|  | T120min | 34.403 | 0.568 | 1.65% |
|  | T240min | 69.027 | 0.746 | 1.08% |
| Mass increase vs T0 (%) | T30min | 0.201 | 0.018 | 8.78% |
|  | T60min | 0.377 | 0.022 | 5.89% |
|  | T120min | 0.752 | 0.034 | 4.59% |
|  | T240min | 1.509 | 0.065 | 4.30% |

**Gravity administration**

- Infusion set Intrafix Safeset, BBraun

|  |  | mean | standard deviation | variation coefficient |
| --- | --- | --- | --- | --- |
| DEHT concentration (mg/dm2) | T30min | 0.06 | 0.03 | 50.03% |
|  | T60min | 0.19 | 0.10 | 54.46% |
|  | T120min | 0.46 | 0.23 | 50.23% |
|  | T240min | 0.96 | 0.47 | 48.93% |
| Mass of simulant collected (g) | T30min | 4.66 | 1.85 | 39.72% |
|  | T60min | 10.29 | 3.50 | 34.03% |
|  | T120min | 27.23 | 7.33 | 26.90% |
|  | T240min | 54.21 | 9.07 | 16.72% |
| Mass increase vs T0 (%) | T30min | 0.10 | 0.04 | 41.24% |
|  | T60min | 0.21 | 0.08 | 38.50% |
|  | T120min | 0.58 | 0.15 | 26.61% |
|  | T240min | 1.16 | 0.18 | 15.86% |

- Infusion set A64, Carefusion

|  |  | mean | standard deviation | CV |
| --- | --- | --- | --- | --- |
| Mass of simulant collected (g) | T30min | 10.02 | 2.92 | 29.12% |
|  | T60min | 19.86 | 6.05 | 30.47% |
|  | T120min | 45.04 | 9.09 | 20.19% |
|  | T240min | 88.65 | 3.28 | 3.70% |
| Mass increase vs T0 (%) | T30min | 0.13 | 0.04 | 29.39% |
|  | T60min | 0.25 | 0.08 | 30.82% |
|  | T120min | 0.57 | 0.12 | 20.70% |
|  | T240min | 1.12 | 0.05 | 4.07% |
